# Promoting self-management through adherence among heart failure patients discharged from rural hospitals: a study protocol

**DOI:** 10.12688/f1000research.5998.2

**Published:** 2015-05-07

**Authors:** Lufei Young, Sue Barnason, Van Do

**Affiliations:** 1 College of Nursing-Lincoln Division, University of Nebraska Medical Center, Lincoln, NE 68588-0220, USA; 2 Department of Health Services Research & Administration College of Public Health, University of Nebraska Medical Center, Omaha, NE 68198-4350, USA

**Keywords:** heart failure, self-management, patient activation, adherence

## Abstract

**Background **Heart failure is one of the most prevalent chronic conditions in adults, leading to prolonged morbidity, repeated hospitalizations, and placing tremendous economic burden on the healthcare system. Heart failure patients discharged from rural hospitals, or primarily critical access hospitals, have higher 30-day readmission and mortality rates compared to patients discharged from urban hospitals. Self-management improves heart failure patients’ health outcomes and reduces re-hospitalizations, but adherence to self-management guidelines is low. We propose a home based post-acute care service managed by advanced practice nurses to enhance patient activation and lead to the improvement of self-management adherence in heart failure patients discharged from rural hospitals.

**Objective **This article describes the study design and research methods used to implement and evaluate the intervention.

**Method** Our intervention is a 12-week patient activation (Patient AcTivated Care at Home [PATCH]) to improve self-management adherence. Patients were randomized into two parallel groups (12-week PATCH intervention + usual care vs. usual care only) to evaluate the effectiveness of this intervention. Outcomes were measured at baseline, 3 and 6 months.

**Discussion**

This study aimed to examine the effectiveness of a rural theory based, advance practice nurse led, activation enhancing intervention on the self-management adherence in heart failure patients residing in rural areas. Our expectation is to facilitate adherence to self-management behaviors in heart failure patients following discharge from rural hospitals and decrease complications and hospital readmissions, leading to the reduction of economic burden.

**Clinical Trial Registration Information:** ClinicalTrials.gov; https://register.clinicaltrials.gov/ NCT01964053

## Study rationale

Heart failure is one of the most prevalent chronic diseases among the adult population ^1^ and hospitalizations account for the majority of costs related to heart failure treatment ^2^. Rural hospitals had higher 30-day readmission rates for heart failure patients than urban hospitals (28% vs. 25%) ^3, 4^ ( https://ruralhealth.und.edu/pdf/umrhrc_finalreport1110.pdf). Self-management is key to improving heart failure patients’ health outcomes ^5^ and reducing re-hospitalizations ^6, 7^. Non-adherence to self-management guidelines accounted for 50% of hospital readmissions in heart failure patients ^8, 9^.

Compared to urban residents, patients in rural communities face greater challenges in managing their heart failure ^10^. Difficulties include lack of local cardiac services and heart failure specialists ^3, 10^, lack of heart failure specific self-management guidance from providers ^11, 12^, less hospital discharge education at critical access hospitals, lack of follow-up by providers ^13, 14^, poor communication between the patient and providers, difficulty in traveling long distances for follow-up appointments and associated problems (time, fatigue, and cost) ^11^, and feeling isolated and unsupported ^15, 16^. Despite these identified needs, effective programs to support heart failure patients in managing this complex, chronic condition in rural communities have not been reported ^10^. In addition, there is lack of reimbursement for programs that promote heart failure patients engaging in self-management behaviors over time. Innovative programs, such as the proposed PATCH program, are needed to assist heart failure patients’ self-management adherence.

The effective interventions to improve adherence to heart failure self-management behaviors are primarily disease management programs ^17^ which require intensive resources and are mainly delivered in urban areas with comprehensive medical care centers. The limitations of existing interventions to promote self-management adherence in rural heart failure patients include: lack of theoretical guidance for the development of a rural-based intervention ^18, 19^, unclear mechanism of intervention ^8, 17, 20^, and reliance on self-report measures of self-management adherence ^21– 23^.

Our study will fill the gap of knowledge and evidence existing in the current literature about self-management interventions by: 1) identifying and appraising new intervention mechanisms to improve self-management behaviors; 2) testing the feasibility and efficacy of a rural theory-based intervention designed to assist rural heart failure patients in managing their chronic condition; and 3) evaluating the use of biomarkers (i.e., brain natriuretic peptide [BNP] and sodium concentration collected from a spot urine sample) to assess the adherence of self-management behaviors.

## Conceptual framework

**Figure 1.**
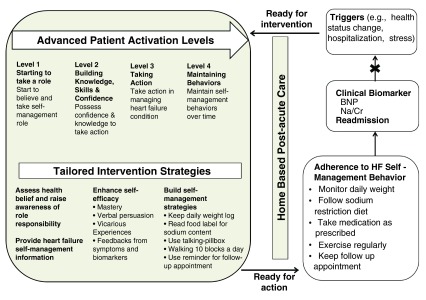
Patient Ac Tivated Care at Home Model: PATCH.

Self-management adherence is defined as the ability to follow and engage in self-management behaviors recommended in heart failure treatment guidelines (e.g., monitor daily weight, follow a restricted sodium diet, take medication as prescribed, exercise regularly, and keep follow-up appointments) ^24^. We have proposed the patient activation intervention PATCH (Patient AcTivated Care at Home Model) for this study based on components of Lorig’s chronic disease self-management model ^25^, Hibbard’s patient activation theory ^26, 27^, Bandura’s conceptualization of self-efficacy ^28^, and Long and Weinert’s rural nursing theory ^19^ ( Figure 1). According to Long and Weinert’s rural nursing theory, rural patients are more likely to accept help and care during times of crisis ^19^. Therefore, the intervention is triggered by the patient’s hospitalization and initiated during their hospital stay when they feel most vulnerable and receptive to the idea of making behavioral change to avoid readmission. Rural patients’ belief about self-reliance (responsibility for one’s own care) supports the use of Hibbard’s patient activation theory ^26^.

In summary, the goal of the PATCH intervention is to increase adherence to self-management behaviors, leading to improved clinical biomarkers (BNP and urine sodium concentration) and fewer hospital readmissions that are considered to be threats to their health beliefs (health is to work, be productive and function in one’s own role) ^19^. Our central hypothesis, based upon our preliminary data, is that patients with higher activation levels, as assessed by the Hibbard patient activation measure, will have significantly better self-management adherence. Given the significant challenges of managing heart failure patients in rural settings, it is essential to examine the feasibility, acceptability, and size of the effects of PATCH on adherence to self-management behaviors and readmissions.

We test our intervention with the following aims:

**Aim 1. To evaluate the immediate and extended effects of the patient activation intervention on self-management adherence**, we measure adherence using clinical biomarkers and self-report of self–management behaviors. Our working hypothesis (H _1_) is that subjects in the intervention group have better self-management adherence than the usual care group over time (3 and 6 months).

**Aim 2. To evaluate the immediate and extended effects of the patient activation intervention on the specific health outcome**, we measure hospital readmission rates. Our working hypothesis (H _2_) is that subjects in the intervention group have lower readmission rate than the usual care group over time (30 days, 3 and 6 months).

**Aim 3. To evaluate the mechanism of the patient activation intervention.** Our working hypothesis (H _3_) is that the scores on self-management knowledge, self-efficacy for self-management, patient activation, and self-management strategies in the intervention group are higher than the usual care group at the end of the intervention (3 months) when the maximum difference for each variable is expected.

**Aim 4. To evaluate the feasibility of the PATCH intervention** for a future larger clinical trial, which includes evaluation of enrollment (recruitment efficiency, attrition, problems and solutions), intervention fidelity (delivery, receipt, enactment), data collection, subject acceptability of the intervention, and estimation of effect sizes for sample size determination.

## Methods/design

### Study settings

**Figure 2.**
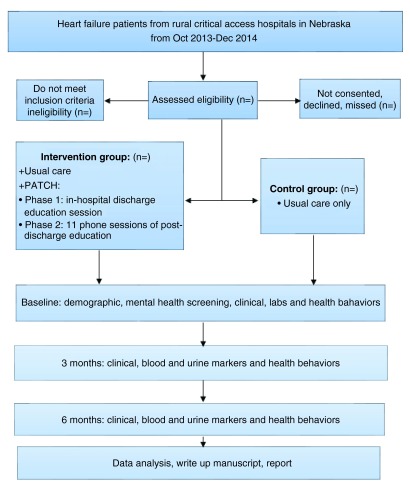
Promoting Self-management through Adherence among Heart Failure Patients Discharged from Rural Hospitals- Flow Chart of Study Design.

Study participants were recruited and enrolled between October 2013 and December 2014 from two rural critical access hospitals. The principal investigator and research assistants who have ethical access at each study site were responsible to identify the potential participants, screen for eligibility and recruitment ( Figure 2).

### Study design

This study is a prospective, two-group, randomized experimental design with three data collection points (baseline, 3 months and 6 months). Heart failure patients discharged from the rural hospitals were randomized into two groups: the intervention or control groups.

1. **Control group** received only usual care. Usual care refers to the standardized care received after hospital discharge, including the written discharge information and the scheduled follow-up doctor appointments. Standardized discharge instructions, as recommended by CMS and the Joint Commission, include: activity level, diet, discharge medications, weight monitoring, and what to do if symptoms worsen.
2. **Intervention group** received usual care and the 12 weeks of PATCH intervention. The intervention comprised of two phases in which the in-hospital discharge education session was followed by 12 weeks of post-discharge education sessions delivered by telephone.

### Sample size

Because this is a preliminary study, sample size was estimated for two-sided statistical tests using a liberal α level of .10. For Aim 1, a repeated-measures ANOVA with an average between-group difference of Cohen’s f=.25 (a medium effect) and a within-subject correlation of ≤.6 would require 41 patients per group to have power=.80. With this sample size, a z-test of independent proportions would have power=.79 if the group proportions meeting guidelines differed by approximately .25, a value reached or exceeded by medication, diet, and weighing adherence in most of the intervention trials reviewed ^29^. The sample of 82 also would be large enough to estimate proportions ± .07–.13 with 90% confidence (the precision depends on the value of the proportion and whether it was calculated within-group or for the entire sample). Allowing for 15% attrition, 48 patients per group (total N=96) are recruited.

### Participants

***Inclusion criteria.*** Patients were eligible for the study if they: 1) were age 21 or older; 2) had heart failure as one of their discharge diagnoses; 3) had New York Heart Association (NYHA) class II to IV ( http://www.heart.org/HEARTORG/Conditions/HeartFailure/AboutHeartFailure/Classes-of-Heart-Failure_UCM_306328_Article.jsp) or had NYHA class I symptoms and at least one other heart failure-related hospitalization or emergency department visit in the year prior to the study; 4) were discharged to home; 5) passed a mini-cognitive screen ^30^; 6) understood English; and 7) had access to a phone.

***Exclusion criteria.*** Patients were not eligible for the study if they: 1) had depressive symptoms (received a score of 3 or above on the Patient Health Questionnaire-2 (PHQ-2)) ^31^ ( http://www.commonwealthfund.org/usr_doc/PHQ2.pdf); 2) had documented medical diagnosis or diagnostic evidence of liver cirrhosis; 3) had documented medical diagnosis or diagnostic evidence of renal failure defined as serum creatinine greater than 2.0mg/dl; and 4) had documented medical diagnosis or diagnostic evidence of end stage and/or terminal illness (e.g. cancer) affecting their abilities to perform self-management behaviors.

## PATCH intervention

The intervention group received usual care and the PATCH intervention. The intervention was comprised of two phases in which the in-hospital discharge education session was followed by 12 weeks of post-discharge education sessions delivered by telephone. The telephone delivery mode was a reliable method to reach patients living in rural counties where internet service was often unreliable and costly. In addition, telephone contact was preferred by many elderly patients because of the complexity of navigating and manipulating other communication platforms ^32^.

In addition to the complexities, burden and costs, other telehealth delivery modalities presented the following limitations: 1) challenge with recruitment and retention; 2) wide range of variation in intervention administration (i.e., various single dose strength and dosing frequency); and 3) recruitment bias. Previous web-based behavioral interventions reported having low recruitment rate (<11%) ^33^, high attrition rate (>65%) ^34^, and inconsistent intervention administration ^33^. Compared to non-participants, the participants of web-based interventions were predominantly white ^34^, younger ^34, 35^, well-educated ^34, 35^, with greater engagement in seeking health information ^36^ and fewer risk factors (e.g., smoking, obesity) ^35^. Previous studies indicate participants of an intervention study delivered by internet or other similar methods are more likely to have higher baseline activation level and have already engaged in self-management behaviors, leaving little to no room for the intervention to work. It has been reported that the barriers for elderly HF patients using advanced interactive technology are low energy from chronic fatigue ^37^ and inadequate health and computer literacies ^38, 39^. Thus the telephone platform is used to deliver the intervention so that we can reach the HF patients who are more likely to have low levels of patient activation and refuse to participate in the study if the intervention delivery methods are perceived to be too complex or burdensome.

During **Phase I (in-hospital discharge education session)**, the intervention was delivered in the hospital to capture a “teachable moment” when patients had recently experienced deteriorated health and recognized the need to better manage their heart failure. The intervention was focused on assessing the patient’s intent and readiness to assume a self-management role or encouraging the patient to assume this role (patient activation level 1) and building knowledge, skills and confidence specific to areas of knowledge deficit identified by the patient (patient activation level 2). The teaching materials included: 1) an educational workbook developed by Dr. Darren DeWalt at the Cecil G. Sheps Health Services Research Center for heart failure patients ( http://www.nchealthliteracy.org/comm_aids/Heart_Failure_Intervention_eng_v1.pdf), 2) the Agency for Healthcare Research and Quality (AHRQ) guide book for patients discharged from hospitals ( http://www.ahrq.gov/qual/goinghomeguide.pdf) and 3) the personal stories about living with heart failure posted on the American Heart Association webpage ( http://www.heart.org/HEARTORG/Conditions/HeartFailure/HeartFailureToolsResources/Heart-Failure-Personal-Stories_UCM_306386_Article.jsp). The overall goal was to establish the initial patient-provider relationship and encourage patients to take an active role in self-management. At discharge, each participant from the intervention group received an intervention toolkit containing the heart failure self-management workbooks, an electronic talking pillbox and a digital scale.

During **Phase II** ( **post-discharge phone education sessions**), a total of 11 phone contacts were made with the patient (twice a week for the first 2 weeks, once a week for weeks 3–6, and every other week for weeks 7–12). Each session focused on 1–2 topics and confirmed the patient’s understanding of the knowledge and skills delivered during their hospital stay. The goals for the **Phase II** intervention were to establish a therapeutic patient-provider relationship and to monitor and reinforce self-management behaviors. Each session started with an informal assessment of the patient’s activation level and the intervention strategies were modified based on the results. The length of the intervention and number of sessions were similar to Wolever’s study that showed effects of a telephone delivered patient activation intervention on the improvement of self-management behaviors in type 2 diabetic patients ^40^.

## Outcome measures and data collection

Table 1 describes the outcome variables specified in the study aims, the study instruments used, their psychometric characteristics, and data collection points.

**Table 1.** Data Collected in the Study.

VARIABLE	MEASURES AND DATA COLLECTION TIME POINTS	Data analysis
BASELINE SCREENING (PRIOR TO ENROLLMENT)		
Cognition	Mini-cog Screen: to screen for cognitive impairment in older adults. Score ranges from 0 for cognitive impairment to 3 for no impairment ^30^	
Depression	Patient Health Questionnaire-2 (PHQ-2): to screen for depression, providing a 0 to 6 severity score (cut score of 3 for depression) ^31^ ( http://www.commonwealthfund.org/usr_doc/PHQ2.pdf)	
BACKGROUND VARIABLES (BASELINE)		
Demographic and Clinical variables:	Demographic and Clinical Variables Tool: demographic (e.g., age, gender, race/ethnicity) and clinical data (e.g., comorbidity, ejection fraction, NYHA score, medications, medication changes, previous admissions).	T-test Chi-square
Monitoring daily weight	One question on the Follow-up Data Collection Survey: How many days per week do you weigh yourself?	T-test
Following low sodium diet	One question on the Follow-up Data Collection Survey: How many days per week do you follow a low-sodium diet?	T-test
Taking prescribed medications	• Medical Outcome Study (MOS) Medication Adherence Scale: During the past 7 days (including last weekend), how many days have your missed taking ANY of your doses? • Medication Adherence in Heart Failure Patients ^41^: 32-items measuring factors influencing adherence to the prescribed medication regimen.	T-test
Exercise regularly (Physical Activity)	• Physical activity is measured using the GT3X: ActiGraph accelerometer ^42^ Data obtained include average daily activity counts, average expended energy (kcal/kg/day, Estimated Energy Expenditure [EEE]), and average activity intensity (kcal/day) • One question on the Follow-up Data Collection Survey: How many days per week do you exercise (e.g., walking)?	T-test
Attending the scheduled appointment	One question on the Follow-up Data Collection Survey: In the last month, did you go to the scheduled follow-up appointment with your primary care provider (or heart doctor) after dismissal from the hospital? Yes or No	Chi-square
Clinical Biomarkers	Serum BNP level: the whole blood specimen is collected and BNP Fragment (nt-proBNP 8–29) ELISA (Triage® BNP test, ALPCO Diagnostics, Inc. Salem, NH)	T-test
	Urine sodium (Na) and creatinine (Cr) level: the urine sample is used to determine the urine Na and Cr levels ( http://www.questdiagnostics.com/testcenter/BUOrderInfo.action?tc=8514X &labCode=SJC)	T-test
OTHER FOLLOW-UP DATA COLLECTION SURVEY ITEMS (3, AND 6 MONTHS)		
Re-admission rate	• Follow-Up Data Collection Survey Tool (i.e, healthcare utilization, work status, current medications) • Medical records to validate healthcare utilization including readmission rates	KM analysis
INTERVENTION COMPONENT MEASURES (BASELINE, 3 MONTHS)		
Self-management Knowledge	Atlanta Heart Failure Knowledge Test (AHFKT-V2) ^43^: 27-item multiple-choice questions to measure HF self-management knowledge. Total scores range from 0 to 27.	Repeated ANOVA (rANOVA)
Self-efficacy	Self-Efficacy for Heart Failure Self-management: Self-care of HF Index Section C, 6 items (questions 17–22). Scores are standardized to range from 0 to 100, with higher scores indicating higher self- efficacy.	rANOVA
Patient Activation	Patient Activation Measure (PAM) ^26^: 13 items with a 5-point Likert response scale. The raw scores are summed and transformed to 0–100 metric (0 = lowest activation level, 100 = highest).	rANOVA
Self-management Strategies	Heart Failure self-management strategies: 29-item Revised Heart Failure Self-Care Behavior Scale (RSCB) ^44^ asks patients to rate how often they performed a behavior in the last few days: on a scale from 0=none of the time to 5=all of the time.	rANOVA

### Statistical methods

A two-sided, alpha level of 0.10 was used to identify trends in the group differences on outcomes. Descriptive statistics are reported at baseline, 3 months and 6 months. Chi-square tests were used to evaluate the difference between proportions. We used t-tests to compare the averages of continuous variable between groups.

Linear mixed model methods are used to compare the groups across the 6-month period, adjusting for baseline levels on the respective outcome. We used ANOVA analysis for repeated measures. Kaplan-Meier method is used for survival analysis to estimate the difference of hospital readmission occurrence between groups.

## Discussion

This study will examine the effectiveness of a rural theory based, advance practice nurse led, activation enhancing intervention on the self-management adherence in heart failure patients residing in rural areas. The findings of this study could fill the gap of knowledge in self-management research in rural heart failure populations.

The long-term goals of this research are to: 1) test this patient activation intervention in other rural patient populations with multiple chronic conditions; 2) develop a rural based patient activation conceptual framework to guide the design and implementation of interventions to promote life-long self-management adherence in rural and underserved communities; and 3) develop a point of care tool kit for heart failure patients to provide timely feedback about their performance in managing their chronic conditions.

Our expectation is to facilitate adherence to self-management behaviors in heart failure patients following discharge from rural hospitals and decrease complications and hospital readmissions, leading to the reduction of economic burden.
