# Supplementary material for: Promoting self-management through adherence among heart failure patients discharged from rural hospitals: a study protocol
Source: F1000Res. 2015 May 7;3:317. Originally published 2014 Dec 30. [Version 2] doi: 10.12688/f1000research.5998.2 (PMC4367517; doi:10.12688/f1000research.5998.2)
Supplement: Peer review history

# Peer review: Referee response for version 1

**DOI:** 10.5256/f1000research.6416.r7608

**Published:** 2015-03-18

**Authors:** Kevin T. Fuji

**Affiliations:** 1 Center for Health Services Research and Patient Safety, Creighton University, Omaha, NE, USA

This is a well-written and well thought-out protocol for an exploratory (or pilot) approach to the use of the PATCH intervention in critical access hospitals. There are just a few additional points of clarification that would further strengthen the paper. 

During Phase II (post-discharge phone education sessions), what happens if patients cannot be reached?  How many attempts are made to reach each patient? At what point of non-contact is the patient excluded from the study?

The authors might consider adding a third column to Table 1 – “Data Analysis” which could describe the data analysis procedures used to analyze each variable. 

In Figure 1, where is the description for “Level 2 Building Knowledge, Skills & Confidence” that matches the other Levels in the figure?

I have read this submission. I believe that I have an appropriate level of expertise to confirm that it is of an acceptable scientific standard.

# Response

**Published:** 2015-04-27

**Authors:** Lufei Young

**Affiliations:** University of Nebraska Medical Center, USA

*"During Phase II (post-discharge phone education sessions), what happens if patients cannot be reached?  How many attempts are made to reach each patient? At what point of non-contact is the patient excluded from the study?*"

Author Response: During Phase II, we tried to reach patients 3 times. If we fail to reach patients after 3 attempts, the patient will be excluded from the study. Fortunately, we haven’t had to exclude any patient during Phase II

*"The authors might consider adding a third column to Table 1 – “Data Analysis” which could describe the data analysis procedures used to analyze each variable."*

Author Response: Author added analysis procedures will be used for each variable as suggested.

*"In Figure 1, where is the description for “Level 2 Building Knowledge, Skills & Confidence” that matches the other Levels in the figure?"*

Author Response: We added the description for level 2 of patient activity level in the figure.

# Peer review: Referee response for version 1

**DOI:** 10.5256/f1000research.6416.r7154

**Published:** 2015-01-28

**Authors:** Huo Yong

**Affiliations:** 1 Department of Cardiology, Peking University Health Science Center, Beijing, China

This manuscript by Young *et al. *does an excellent job aiming to propose an intervention and evaluation of it for self-management among heart failure patients discharged from rural hospitals. With the prevalence of heart failure, self-management of patients plays a critical role in their improvement of quality of life, relief of exacerbation of symptoms and readmission to hospitals. Patients discharged from rural hospitals especially need education and guidance to improve adherence. The authors tried to solve this needs and described in detail the innovative intervention of a 12-week patient activation (Patient AcTivated Care at Home [PATCH]). And this paper is well-organized in clarifying the rational and design of the study to evaluate feasibility and efficacy of PATCH. Moreover, the authors showed us a vision of implementation of this intervention. 

The genesis of this study based on solid nursing and patients’ activation theories. And a prospective, two-group, randomized experimental design with three data collection points (baseline, 3 months and 6 months) were chosen to assess the feasibility and efficacy of the intervention. Sample size was carefully estimated. Also, different measures on biomarkers, outcomes and self-management were designed. In order to prevent the possible cognitive or psychotic confounding factors, simple screening tests were required to conduct prior to enrollment. The article was well constructed, the study was well designed, and interpretation was prudent.

However, there are some comments and suggestions for authors to consider. First, is it practical for a rural heart failure patient to complete so many questions on measurements of self-management knowledge, self-efficacy, patients’ activity and strategies at one visit? Suggest simplified measurable scales. Second, for measurements of adherence of self-management, only data at 3 months were collected. Will it help to analyze extended effects of the intervention by record data at 6 months?

Finally, this work is a good reminder for all the cardiologists to pay more attention to the management and education of heart failure patients. The novel intervention of PATCH might acts as an example to help more and more heart failure patients.

I have read this submission. I believe that I have an appropriate level of expertise to confirm that it is of an acceptable scientific standard, however I have significant reservations, as outlined above.

# Response

**Published:** 2015-04-27

**Authors:** Lufei Young

**Affiliations:** University of Nebraska Medical Center, USA

*"First, is it practical for a rural heart failure patient to complete so many questions on measurements of self-management knowledge, self-efficacy, patients’ activity and strategies at one visit? Suggest simplified measurable scales.*"

Author Response: We greatly appreciate your suggestion and will use objective measures for activity level, adherence and knowledge in the larger scale study.

*"Second, for measurements of adherence of self-management, only data at 3 months were collected. Will it help to analyze extended effects of the intervention by record data at 6 months?"*

Author Response: This is a feasibility study aimed to examine the magnitude of intervention on variable of interest. To determine the mechanism of intervention, the group comparison was made at 3 months when the 12-week intervention was just completed so the maximal differences would be expected between intervention and control groups in self-management knowledge, self-efficacy for self-management, patient activation, and self-management strategies. Another reason to assess 3-month only is to reduce subject burden. The population studied is characterized as elderly living with heart failure and other debilitating chronic conditions.  Fatigue and functioning declination are primary symptoms in this population.

***"**Finally, this work is a good reminder for all the cardiologists to pay more attention to the management and education of heart failure patients. The novel intervention of PATCH might acts as an example to help more and more heart failure patients."*

Author Response: We really appreciate your recognition of the practice implication of this study. Thank you so much.

# Peer review: Referee response for version 1

**DOI:** 10.5256/f1000research.6416.r7376

**Published:** 2015-01-19

**Authors:** Pascale Salameh

**Affiliations:** 1 Faculty of Public Health, Section II, Lebanese University, Beirut, Lebanon

This is an excellent work, well justified and adequately addressed. However, some points should be met:

**Study design: **References are needed for interventions. More details are particularly needed for the major PATCH intervention.

**Figure 2: **Why not add the number of patients for each step, since the study is already over?

**Statistical analysis: **Why adjust over baseline value of the measure in question only? Adjustment over other baseline variables may also be needed in case randomization did not succeed to equilibrate them. For readmission, we think that Cox regression model would be more adequate than KM analysis.

**One more point:** In several locations in the manuscript, the authors say it is an exploratory work. Why? What is lacking?

I have read this submission. I believe that I have an appropriate level of expertise to confirm that it is of an acceptable scientific standard.

# Response

**Published:** 2015-04-27

**Authors:** Lufei Young

**Affiliations:** University of Nebraska Medical Center, USA

***"**Study design: References are needed for interventions. More details are particularly needed for the major PATCH intervention."*

Author Response: Thank you for your suggestion. We added the reference and more detail for our intervention.

***"**Figure 2: Why not add the number of patients for each step, since the study is already over?"*

Author Response: Our study is still collecting data for 3 months and 6 months so we haven’t gotten the data for these steps yet. Moreover, given that our manuscript is a research protocol, we think the data result is not mandatory

***"**Statistical analysis: Why adjust over baseline value of the measure in question only? Adjustment over other baseline variables may also be needed in case randomization did not succeed to equilibrate them. For readmission, we think that Cox regression model would be more adequate than KM analysis."*

Author Response: Thank you for valuable comment. We will adjust all relevant baseline variables in case randomization did not succeed to attenuate the differences between groups. The aim of the study is to examine the effects of 12-week patient activation enhancing intervention on self-management adherence. Identifying the potential predictors of readmission and assessing how intervention affecting the identified predictors of readmission are not the main aim of the study, which was not designed to examine predictors of readmission. Therefore, Kaplan-Meier method is preferred due to its simplicity. In the future manuscript, we may use Cox regression model with multiple predictors for readmission if our survival analysis need to adjust for the other risk factors.

*"One more point: In several locations in the manuscript, the authors say it is an exploratory work. Why? What is lacking?"*

Author Response: The purpose of this study is to evaluate the feasibility of the PATCH intervention and gather initial data to support a larger investigation. But in order to avoid misunderstanding, the author will remove the word, “exploratory" as suggested.
